# Screening Tests for the Rapid Detection of Diarrhetic Shellfish Toxins in Washington State

**DOI:** 10.3390/md11103718

**Published:** 2013-09-30

**Authors:** Bich-Thuy L. Eberhart, Leslie K. Moore, Neil Harrington, Nicolaus G. Adams, Jerry Borchert, Vera L. Trainer

**Affiliations:** 1 NOAA, Northwest Fisheries Science Center, Marine Biotoxins Laboratory, 2725 Montlake Blvd. E., Seattle, WA 98112, USA; E-Mails: leslie.moore@noaa.gov (L.K.M.); nicolaus.adams@noaa.gov (N.G.A.); vera.l.trainer@noaa.gov (V.L.T.); 2 Jamestown S’Klallam Tribe, 1033 Old Blyn Highway, Sequim, WA 98392, USA; E-Mail: nharrington@jamestowntribe.org; 3 Food Safety and Shellfish Program, Washington State Department of Health, 7171 Clearwater Lane, Olympia, WA 98504, USA; E-Mail: jerry.borchert@doh.wa.gov

**Keywords:** diarrhetic shellfish poisoning (DSP), diarrhetic shellfish toxins (DSTs), okadaic acid, rapid screening test, LC-MS/MS, protein phosphatase 2A (PP2A), enzyme-linked immunosorbent assay (ELISA), Jellett rapid test, Puget Sound

## Abstract

The illness of three people due to diarrhetic shellfish poisoning (DSP) following their ingestion of recreationally harvested mussels from Sequim Bay State Park in the summer of 2011, resulted in intensified monitoring for diarrhetic shellfish toxins (DSTs) in Washington State. Rapid testing at remote sites was proposed as a means to provide early warning of DST events in order to protect human health and allow growers to test “pre-harvest” shellfish samples, thereby preventing harvest of toxic product that would later be destroyed or recalled. Tissue homogenates from several shellfish species collected from two sites in Sequim Bay, WA in the summer 2012, as well as other sites throughout Puget Sound, were analyzed using three rapid screening methods: a lateral flow antibody-based test strip (Jellett Rapid Test), an enzyme-linked immunosorbent assay (ELISA) and a protein phosphatase 2A inhibition assay (PP2A). The results were compared to the standard regulatory method of liquid chromatography coupled with tandem mass spectroscopy (LC-MS/MS). The Jellett Rapid Test for DSP gave an unacceptable number of false negatives due to incomplete extraction of DSTs using the manufacturer’s recommended method while the ELISA antibody had low cross-reactivity with dinophysistoxin-1, the major toxin isomer in shellfish from the region. The PP2A test showed the greatest promise as a screening tool for Washington State shellfish harvesters.

## 1. Introduction

Diarrhetic shellfish poisoning (DSP) is an illness in humans caused by the ingestion of shellfish contaminated by diarrhetic shellfish toxins (DSTs), including okadaic acid (OA) and the dinophysis toxins (DTXs), which are lipophilic toxins produced by dinoflagellates in the genera *Dinophysis* and *Prorocentrum* [1,2,3]. OA and its analogs (DTX-1, DTX-2 and DTX-3) are acid polyethers that inhibit serine/threonine protein phosphatase activity by binding to its receptor site, resulting in a rapid increase of phosphorylated proteins [4,5,6]. They are the only toxins of the DSP complex with diarrheagenic effects in mammals [7]. Diarrhetic shellfish poisoning symptoms include diarrhea, nausea, vomiting, and abdominal pain starting 30 min to a few hours after ingestion of the toxic shellfish with complete recovery within three days [8]. Tumor-promoting, mutagenic and immunosuppressive effects shown in animals to be associated with DSTs have not yet been confirmed in humans [7] however several studies suggest that chronic exposure may increase the risk of gastrointestinal cancers [9,10,11]. DSP events had been suspected, but not confirmed in the U.S. until recently.

**Figure 1:**
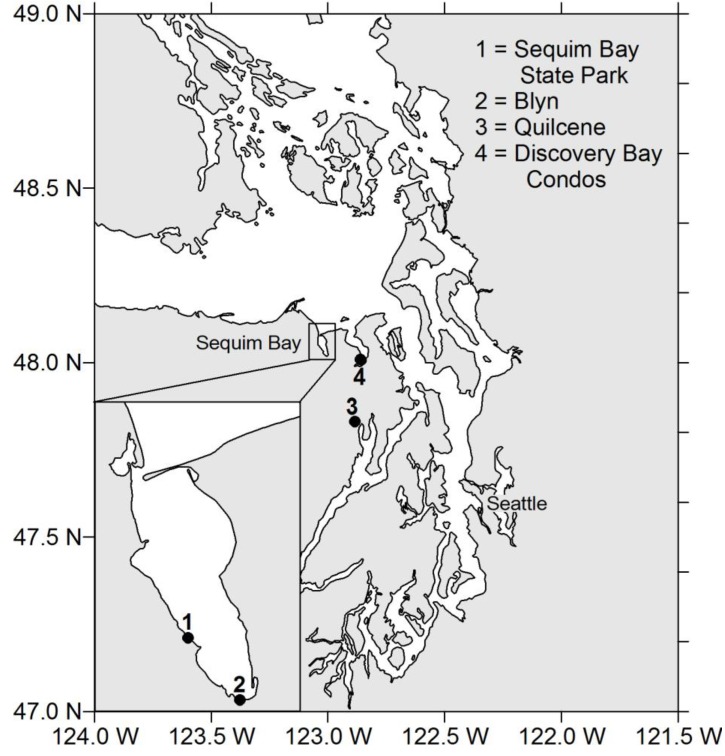
Locations of sites in Sequim Bay and Puget Sound, Washington State where samples were collected for method comparisons and matrix effect tests.

Three DSP cases were reported on 29 June 2011 in the U.S. Pacific Northwest from the consumption of mussels collected from a pier at Sequim Bay State Park (Figure 1). Family members aged 2, 5 and 45 years developed symptoms 4, 7, and 14 h, respectively, after consuming 8–15 mussels [12]. Diarrhetic shellfish poisoning symptoms that were exhibited by the individuals included vomiting, diarrhea, body aches, fever and chills. Blue mussels collected within a few days of the illnesses were found by liquid chromatography tandem mass spectrometry (LC-MS/MS) analysis to contain levels of DSTs 2–10 times the action level of 16 μg OA equiv./100 g shellfish tissue. This finding prompted product recalls and the closure of recreational and commercial shellfish harvesting from Sequim Bay.

Additionally, in July–August 2011, 62 people suffered from DSP in British Columbia, Canada. These illnesses were traced to the ingestion of Pacific coast mussels and were the first reports of DSP in western Canada [13]. Almost 14,000 kg of product was recalled. Although the presence of *Dinophysis* in Pacific Northwest coastal waters dates back many years [14], these events represented the first time illnesses were reported in conjunction with DST levels deemed hazardous to human health.

During the summer of 2012, a comprehensive analysis of DSTs was performed in several shellfish species collected from numerous sites throughout Puget Sound [15]. Detection of DSTs by LC-MS/MS above the regulatory action level resulted in widespread harvest closures of California mussels, varnish clams, manila clams and Pacific oysters [15]. In addition, the first ever closure due to DSTs on the Pacific coast of Washington State occurred at Ruby Beach in August 2012 [15]. These observations confirm that DSTs are a widespread problem of serious consequence to recreational, commercial and subsistence shellfish harvesting in Washington State.

The mouse bioassay had been the official method for detection of lipophilic toxins in shellfish until recent years, when the European Union (EU) Commission adopted and enforced LC-MS/MS as the regulatory method [16]. This regulation also states that alternative or complementary methods may be used as long as the public is equally well protected and that method performance criteria stipulated by the EU guideline are met. In the U.S., the action level for total DSTs (*i.e.*, esterified plus non-esterified: OA + DTXs) is 16 µg OA equiv./100 g shellfish, established by the U.S. Food and Drug Administration (FDA). Detection of DSTs by LC-MS/MS has been used for public health protection in the State of Washington since 2012 but this method is time consuming and expensive. Hence, there is a need for rapid testing that is relatively simple and can be performed quickly at remote sites to provide early warning of DST events. Rapid screening tests would allow growers to test a “pre-harvest” shellfish sample at an onsite laboratory, thereby preventing harvest of toxic product that would later need to be destroyed or recalled.

Currently, there are several commercially available rapid screening test kits. These kits are based on functional action of toxins on their receptors (e.g., protein phosphatase 2A isolated from human red blood cells) or structural recognition of a common epitope (e.g., antibody-based assays, termed enzyme-linked immunosorbent assay or ELISA). Here we describe the analysis of tissue homogenates from several shellfish species collected in the summer of 2012. Samples were collected from two sites in Sequim Bay, WA: Sequim Bay State Park on the western shore and Blyn in the south (Figure 1) and other sites throughout Puget Sound (see Figure 1 in [15]). Three rapid screening methods were used and the results were compared to those obtained from the standard LC-MS/MS regulatory method.

## 2. Results

### 2.1. Analysis of DSTs by LC-MS/MS

**Figure 2:**
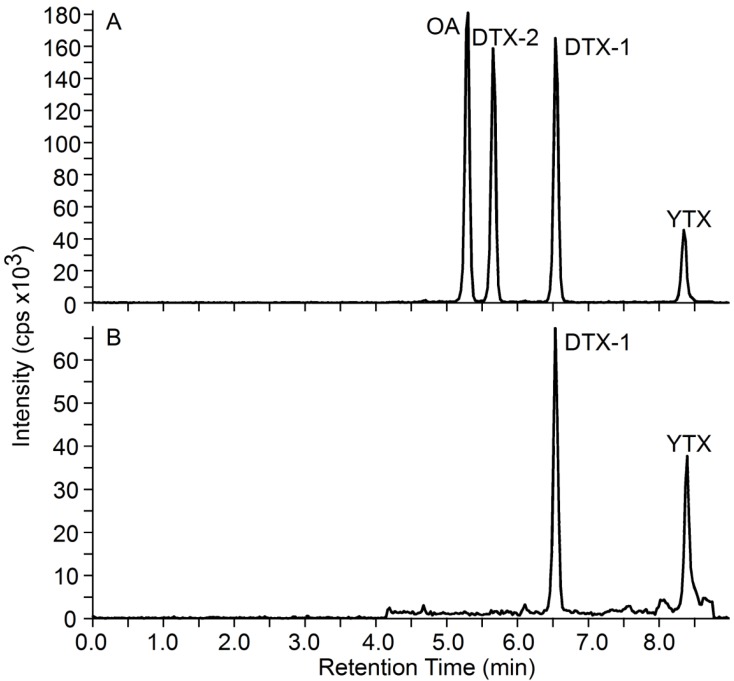
LC-MS/MS chromatograms. Retention times are shown in parenthesis. (**A**) certified reference standards okadaic acid (OA) (5.31 min), DTX-2 (5.67 min), DTX-1 (6.56 min) and yessotoxin (YTX, 8.37 min); (**B**) DTX-1 (6.55 min) and YTX (8.41 min) in a hydrolyzed blue mussel extract from Sequim Bay, WA, USA.

Figure 2A shows a typical LC-MS/MS chromatogram of OA, DTX-1, DTX-2 and yessotoxin (YTX) standards. Although it is often present in shellfish, YTX is a DST that is not currently monitored for regulatory purposes in Washington State. Dinophysistoxin-1 (detected as both free and acyl ester forms) and YTX were found exclusively in the majority of shellfish samples we analyzed. A representative chromatogram of a hydrolyzed blue mussel extract from Sequim Bay, WA is shown in Figure 2B. Our findings confirmed previous survey of Puget Sound showing DTX-1 to be the primary toxin found in shellfish from Puget Sound and the coast of Washington state [15].

### 2.2. Tissue Matrix Effects

The potential matrix effect from shellfish tissue, resulting in over- or under-estimation of the true concentration of analytes present in the sample, was assessed by testing both hydrolyzed and non-hydrolyzed shellfish extracts at several dilutions by LC-MS/MS. Blue mussel samples were analyzed neat and at two dilutions (50%, 10%) for non-hydrolyzed extracts, and a single dilution (50%) for hydrolyzed extracts (Table 1). The DST value of each diluted sample extract was corrected by its dilution factor and compared to the neat extract.

**Table 1:** Diarrhetic shellfish toxins (DST) concentrations by LC-MS/MS analysis of hydrolyzed and non-hydrolyzed blue mussel extracts at three dilutions. Data are corrected for dilution factors. See Figure 1 for site locations.

Hydrolyzed		DST concentration (μg total OA equiv./100 g shellfish)							
Site	OA	DTX-2	DTX-1	OA	DTX-2	DTX-1	OA	DTX-2	DTX-1
(% extract) ^a^	100	100	100	50	50	50	10	10	10
Quilcene	bd	bd	27.92	bd	bd	30.99	na	na	na
Discovery Bay Condos	bd	bd	25.64	bd	bd	25.61	na	na	na
Discovery Bay Condos	bd	bd	21.84	bd	bd	22.23	na	na	na
Sequim Bay State Park	bd	bd	50.31	bd	bd	46.32	na	na	na
Sequim Bay State Park	bd	bd	19.09	bd	bd	16.91	na	na	na
Sequim Bay State Park	bd	bd	82.87	bd	bd	82.48	na	na	na
Sequim Bay Blyn	bd	bd	37.08	bd	bd	33.62	na	na	na
Sequim Bay Blyn	bd	bd	31.53	bd	bd	29.87	na	na	na
									
**Non-hydrolyzed**		**DST concentration (μg OA equiv./100 g shellfish)**							
Site	OA	DTX-2	DTX-1	OA	DTX-2	DTX-1	OA	DTX-2	DTX-1
(% extract) ^a^	100	100	100	50	50	50	10	10	10
Quilcene	1.33	bd	14.18	bd	bd	13.72	bd	bd	12.86
Discovery Bay Condos	bd	bd	13.53	bd	bd	11.40	bd	bd	10.72
Discovery Bay Condos	bd	bd	11.90	bd	bd	10.40	bd	bd	10.01
Sequim Bay State Park	bd	bd	31.65	bd	bd	25.86	bd	bd	16.84
Sequim Bay State Park	bd	bd	26.56	bd	bd	25.92	bd	bd	17.66
Sequim Bay State Park	bd	bd	44.46	bd	bd	20.60	bd	bd	25.42
Sequim Bay Blyn	bd	bd	20.98	bd	bd	18.70	bd	bd	14.23
Sequim Bay Blyn	bd	bd	20.21	bd	bd	18.53	bd	bd	14.52

^a ^Indicates percentage of original extract. All shellfish extracts at less than 100% are diluted with 100% MeOH, bd = below the analytical limit of detection (OA, DTX-1: 1.25 μg/100 g tissue; DTX-2: 1.00 μg/100 g tissue), na = not analyzed.

Matrix effects were found to be low in hydrolyzed blue mussel extracts where DTX-1 values were approximately 3% higher in the neat sample *versus* corrected values of the 50% diluted sample (Table 1), within the calculated error of LC-MS/MS method. This difference was higher in non-hydrolyzed extracts (12%). However, because toxin concentrations were lower in non-hydrolyzed extracts compared to hydrolyzed extracts, the increased instrument or extraction variability at these lower concentrations could be partially responsible for the difference between 100% and 50% (or 10%).

### 2.3. Comparison of Screening Methods to LC-MS/MS

**Figure 3:**
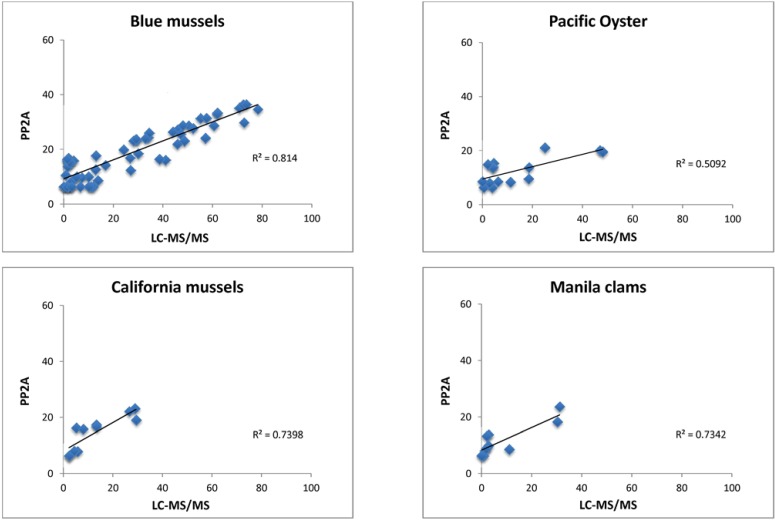
Comparison between LC-MS/MS and PP2A results for total DSTs (μg OA equiv./100 g shellfish extract).

Shellfish samples collected at various locations in Puget Sound and the Pacific coast of Washington State (*n* = 110) were analyzed by PP2A test and compared to LC-MS/MS analysis (Figure 3). These data include the samples shown in Table 2 that were re-extracted for this repeat analysis. The best fit by linear regression was seen with blue mussel (*Mytilus edulis*; *R*^2^ = 0.82; *n* = 63) followed by California mussel (*Mytilus californianus*; *R*^2^ = 0.74; *n* = 11), manila clam (*Venerupis philippinarum*; *R*^2^ = 0.73; *n* = 10), Pacific oyster (*R*^2^ = 0.51; *n* = 15), razor clam (*Siliqua patula*; *R*^2^ = 0.52; *n* = 5; not shown), and littleneck clam (*Leukoma staminea*; *R*^2^ = 0.06; *n* = 6; not shown).

**Table 2:** Methods comparison of LC-MS/MS, PP2A, ELISA and Jellett test strips in samples extracted using the EU method and comparison of LC-MS/MS and Jellett test strips in samples extracted using the Jellett Rapid Test method. DST concentrations are shown (µg total OA equiv./100 g). Positive results: ≥16.0 µg OA equiv./100 g in bold print. Negative result: <16.0 µg OA equiv./100 g in regular print. Jellett rapid test were read visually and noted as positive (+) or negative (−) based on the manufacturer’s method.

	EU Extracts				Jellett Extracts	
Species	LC-MS/MS	PP2A	ELISA	Jellett	LC-MS/MS	Jellett
1. Blue Mussel	**96.5**	**48.9**	**51.8**	−	**39.8**	+
2. Blue Mussel	**63.6**	**38.2**	**52.3**	−	**31.7**	+
3. Blue Mussel	**60.6**	**38.1**	**58.8**	−	**32.4**	+
4. Blue Mussel	**38.7**	**35.7**	**35.7**	−	**18.3**	+
5. Blue Mussel^ a^	**30.3**	**28.7**	**22.6**	−	**17.1**	+
6. Blue Mussel	**36.9**	**36.0**	**63.0**	−	**21.1**	+
7. Blue Mussel	**26.2**	**31.2**	**31.8**	−	12.9	+
8. Blue Mussel	**32.7**	**33.5**	**33.8**	−	15.1	−
9. Blue Mussel	**26.5**	**34.1**	**28.8**	−	13.9	−
10. Blue Mussel	**37.7**	**35.1**	**51.3**	−	**16.9**	+
11. Blue Mussel	**60.5**	**29.4**	**49.3**	−	**26.0**	+
12. Blue Mussel	**28.1**	**33.6**	**25.3**	−	12.7	+
13. Blue Mussel ^a^	**31.5**	**34.5**	**20.5**	−	**18.4**	+
14. Geoduck	<LoQ	<LoQ	10.9	−	<LoQ	−
15. Littleneck Clam	<LoQ	<LoQ	**20.5**	−	2.0	−
16. Manila Clam	**36.1**	**16.9**	**28.8**	−	**16.2**	−
17. Manila Clam	**21.9**	**12.8**	**20.3**	−	13.1	−
18. Pacific Oyster	**25.3**	**16.5**	**67.8**	−	14.5	−
19. Pacific Oyster	6.3	14.7	**27.2**	−	3.0	−
20. Pacific Oyster	3.7	<LoQ	**23.6**	−	2.2	−
21. Pacific Oyster	7.9	13.0	**31.7**	−	2.9	−
22. Pacific Oyster	**37.7**	13.1	**61.3**	−	14.4	−
23. Pacific Oyster	14.2	**26.6**	**32.7**	−	6.5	−

^a ^Extracts of the same sample, LoQ = Limit of Quantification.

**Figure 4:**
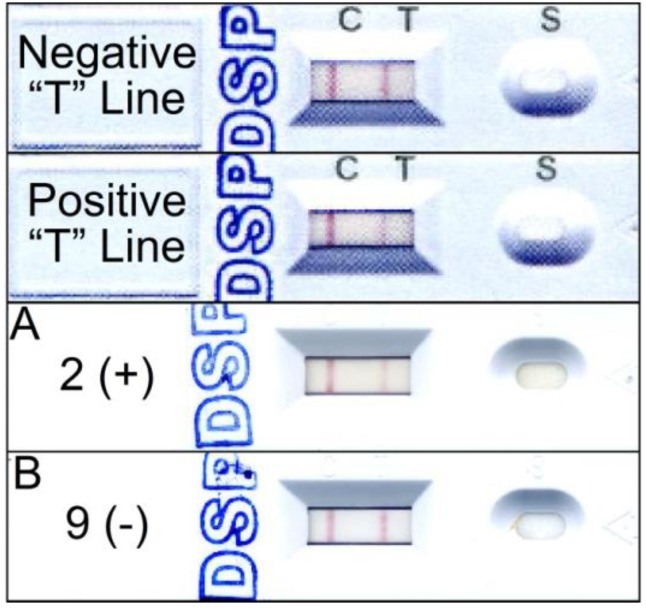
Jellett test strip results for shellfish sample extracts #2 (**A**, positive) and #9 (**B**, negative) from Table 2 compared to reference test strips provided by the manufacturer.

To compare screening methods, shellfish samples (*n* = 23) were extracted by the EU method and analyzed by LC-MS/MS, PP2A, ELISA, and Jellett Rapid Tests (Table 2, Figure 4). There were 5 false positives (samples 15, 19, 20, 21, 23) by ELISA; 2 false negatives (samples 17, 22) and 1 false positive (sample 23) by PP2A while all of Jellett Rapid Test results were negative. However, when this same subset of shellfish extracts were re-tested using the Jellett extraction method and compared to LC-MS/MS results, there were only 6 false negatives (samples 8, 9, 16, 17, 18, 22). The EU method uses a double methanolic extract of shellfish homogenate whereas Jellett manufacturer recommends a single extraction. Comparison of the LC-MS/MS results from samples extracted using the Jellett and EU methods showed that the Jellett method extracted approximately 50% of the DSTs (Table 2).

## 3. Discussion

In Europe, intensified plankton and shellfish monitoring are initiated when cell densities of *Dinophysis* reach 500–1200 cells/L and harvest operations are closed at a threshold level of 5000 cells/L [17]; these precautions would result in almost continuous closures in Washington State where cell numbers of *Dinophysis* at this threshold level are often observed but DSTs in shellfish do not exceed regulatory action levels [15]. Therefore, effective strategies need to be implemented to prevent human illness due to DSP and costly recalls of shellfish product.

Screening tests for DSTs may be useful as an additional management tool for DSP risks. The intention of their use is to ensure that DST-contaminated shellfish are never harvested. In the past, Puget Sound shellfish growers had harvested shellfish and were forced to wait until regulatory testing confirmed shellfish safety, resulting in the disposal of harvested shellfish that were either spoiled or had confirmed toxicity. The success of rapid screening tests is contingent on the ability of these methods to provide an accurate measurement of toxicity. They must be used in a manner that eliminates false negative results and minimizes the number of false positives. For this study, we chose to focus on the PP2A test, a functional assay in which the activity of each toxin isomer corresponds to its ability to inhibit the phosphatase enzyme, in contrast to the ELISA test, a structural assay based upon toxin recognition by an antibody.

Shellfish collected from May 30 to October 2, 2012 throughout Puget Sound for the regulatory management of DSTs, including OA + DTX-1 + DTX-2, quantified in units of OA equivalents (OA equiv.), were extracted, hydrolyzed, and analyzed according to the standard procedure for determination of lipophilic toxins by LC-MS/MS (EU Method). Shellfish were also extracted using the manufacturer’s suggested methods for PP2A and ELISA. Toxin values obtained using these extraction protocols showed no substantial differences to the EU method (data not shown).

The results obtained using LC-MS/MS showed DTX-1 as the major toxin isomer in Puget Sound shellfish (Figure 2). Our results also showed that hydrolysis of blue mussel extracts prior to analysis by LC-MS/MS appeared to eliminate the matrix effects seen in the non-hydrolyzed samples (Table 1). Hydrolysis is required to convert DTX-3, the esterified form of DST toxins present in some shellfish to the parent toxins OA, DTX-1 or DTX-2. Therefore, the hydrolyzed shellfish extracts were used for comparison of toxin values obtained by LC/MS-MS with screening method results.

The first screening method that was assessed in this study was a commercial ELISA that is currently sold in the U.S. by Abraxis (Warminster, PA, USA). Analysis of hydrolyzed shellfish extracts using ELISA in comparison to LC-MS/MS data resulted 5 out of 23 (22%) false positive values. The ELISA overestimated toxin concentrations in hydrolyzed extracts, an interesting finding given that one would expect that the estimated 50% cross reactivity of DTX-1 would result in underestimation of toxin concentrations. The stated LOD for ELISA (10 μg/100 g) combined with the 50% cross-reactivity for DTX-1 and DTX-2 means that when applied to shellfish samples containing only DTX-1 (free and acylated forms), the effective LOD would be 20 μg/100 g, which is above the regulatory action limit. This calls into question the use of the ELISA in Washington State. Other commercial ELISAs, including the “DSP check kit” from Japan, based on the procedure of Usagawa *et al.* [18], have been tested previously [19], however these kits are no longer commercially available. An antibody-based DST test recently has been made available by Neogen (Reveal 2.0 for DSP), however, at the time this study was underway, it was not yet calibrated for use with hydrolyzed samples.

We found that when used as recommended by the manufacturer, the Jellett Rapid Test for DSP gave a high number of false negatives (Table 2), most likely due to incomplete extraction of DSTs by the Jellett extraction method compared to the standard EU method (Table 2). Therefore, improved extraction efficiency is necessary for the Jellett Rapid Test to be used as an accurate screening tool with Washington State shellfish. The need for improving the efficiency of the Jellett Rapid Test extraction method has been recognized by the developers of the test [20]. Once the extraction efficiency is improved, we believe that these test strips will show promise as a screening tool, due to their simplicity of use.

Several trials have been performed with protein phosphatase inhibition assays (PPIA). Different labs have developed their own PPIA tests using absorbance or fluorescence-based endpoint determinations [21,22,23,24,25,26,27,28] with relatively good results. Recently, the first comprehensive single lab validation of a commercial PP2A kit (OkaTest is developed by ZEU-INMUNOTEC, Zaragoza, Spain and distributed in the US by Abraxis LLC) was performed to determine the repeatability, precision, and stability of reagents [29]. This test uses a purified human PP2A that has higher sensitivity than other similar enzymes produced by genetic engineering [30]. The single-lab assessment, in which shellfish were spiked with certified reference solutions and compared to both LC-MS/MS and mouse bioassay results, demonstrated that all reagents were stable for >6 months. Smienk *et al.* [29] determined that the IC_50_ was 1.6 nM for DTX-1 and 1.2 nM for both OA and DTX-2. They reasoned that the lower affinity of the PP2A for DTX-1 would result in underestimation using the commercial PP2A kit compared to LC-MS/MS. Smienk *et al.* [29] also observed that analysis of spiked, hydrolyzed samples gave 146% and 163% recovery for mussel and king scallop, respectively. The authors suggested that the reason for high recovery may be that reference standard materials were certified only for non-hydrolyzed DSTs.

The single lab validation was followed by an interlaboratory validation [31] in which 16 labs were given several species of spiked shellfish and blank samples to analyze. The results indicated that the OkaTest was suitable for quantitative determination of the OA toxins group [31]. In our study, we chose not to replicate this prior investigation, but rather to determine test precision, accuracy, and potential matrix effect in naturally-contaminated shellfish from the Pacific Northwest.

The PP2A test results of the subset of shellfish samples (Table 2) showed 2 false negatives (manila clam and Pacific oyster) and 1 false positive value (Pacific oyster). The 8% false negatives could be reduced through the choice of a lower threshold value (e.g., 12 μg/100 g) for this screening test, which would require that any samples at or above this conservative value be tested by the standard regulatory method. In addition, some sample concentrations predicted by the PP2A test, especially those >60 μg/100 g shellfish, were greatly underestimated using PP2A (Table 2). The manufacturer states that values >35.2 μg/100 g are outside the linear range of the standard curve and suggests that these values be treated as estimates. However, these underestimated high concentrations do not alter the effectiveness of this test as a screening tool.

Some matrix effect was evident using the PP2A test, in that extracts with zero toxin concentrations by LC-MS/MS showed measurable DSTs by PP2A (Figure 3, all panels, see symbols on the *y*-axis). Matrix effects have been observed in past studies where toxin content was overestimated by enzyme assay compared to LC-MS/MS analysis [32]. This effect has been attributed to the presence of compounds such as methanol-soluble lipids that exert an unspecific inhibitory effect on the protein phosphatase [22,29]. Many of these past studies used shellfish homogenates that were spiked with known concentrations of OA and other DSTs. In the future, naturally contaminated samples must be tested, including Pacific Northwest shellfish samples containing varied concentrations of DTX-1 and DTX-3, in order to confirm the effectiveness of PP2A as a screening test.

Harvest closures in the State of Washington are implemented when toxins exceed the regulatory action level [33]. Traditional monitoring programs established by shellfish authorities allow for timely closures in state waters with minimal negative impacts on industry. However, such monitoring programs are not always the most effective at remote sites given the time required for sample shipment and analysis. For example, the Jamestown S’Klallam Tribe has had to purchase and ship shellfish from other locations for their tribal potlatches because of the shellfish harvested from Sequim Bay could not be tested for DSTs in time for the tribal feast. However, in order to use screening tools for rapid assessment of toxins, these methods must be endorsed by the Interstate Shellfish Sanitation Conference (ISSC) for specific applications in federal waters. An example of this process is the ISSC approval of Abraxis shipboard ELISA for paralytic shellfish poisoning (PSP) for the offshore scallop fishery on Georges Bank [34]. Since the Jamestown S’Klallam Tribe is an independent federal entity, they could choose similar monitoring strategies to ensure the safety of shellfish intended for subsistence consumption. The PP2A may provide a safety buffer for the harvest of large amounts of shellfish prior to confirmatory regulatory testing, or may suffice as a tool for specific uses, for example, to maintain an open status of the shellfish beds. Another example of rapid screening test application is the combination of phytoplankton monitoring and ELISA screening tests for domoic acid that are used by the Olympic Region Harmful Algal Bloom (ORHAB) partnership to maintain opening status of razor clam beds. We envision that a combination of phytoplankton monitoring and rapid screening tests might be implemented as a complement to regulatory monitoring, especially at remote sites in Washington State.

## 4. Experimental Section

### 4.1. Shellfish Collection

For comparing LC-MS/MS and the three screening methods, shellfish, including blue mussel (*Mytilus edulis*; *n* = 13), geoduck clam (*Panopea generosa*; *n* = 1), littleneck clam (*Leukoma staminea*; *n* = 1), manila clam (*Venerupis philippinarum*; *n* = 2) and Pacific oyster (*Crassostrea gigas*; *n* = 6), were collected at two locations in Sequim Bay in Washington State (Figure 1) and stored at −20 °C until analysis. Samples for detailed comparison of PP2A and LC-MS/MS were collected from sites throughout Puget Sound and included blue mussel (*n* = 63), California mussel (*n* = 11), manila clam (*n* = 10), Pacific oyster (*n* = 15), razor clam (*n* = 5), and littleneck clam (*n* = 6).

### 4.2. Sample Preparation

Shellfish were rinsed with tap water and opened by cutting the adductor muscles. About 10–20 individual shellfish were pooled to make up at least 100 g of tissue per sample. Samples were drained for a few minutes to remove any excess water and then homogenized in a glass blender for 1 min. The homogenates were stored in ultra-high performance polypropylene copolymer containers at −20 °C until analysis.

### 4.3. Tissue Extraction

The method for shellfish tissue extraction was a modification of the EU-Harmonized Standard Operating Procedure for the Determination of Lipophilic Marine Biotoxins in Mollusks by LC-MS/MS [35]. Briefly, an aliquot of sample homogenate (2.5 g) was accurately weighed into a 50 mL centrifuge tube (BD Falcon, San Jose, CA, USA) and extracted with 12 ml of methanol by vortex mixing for 3 min. The mixture was centrifuged at 2500× *g* for 10 min and the supernatant transferred to a 25 mL volumetric flask. The residual pellet was re-extracted by homogenizing in 10 mL methanol for 1 min with a 10 mm stainless steel OmniProbe High Power Tissue Homogenizer (Kennesaw, GA, USA) followed by centrifugation at 2500× *g* for 10 min. The supernatant was combined with the first extract and brought to 25 mL with methanol. Samples also were extracted using the method specified by the manufacturer of the Jellett Rapid Test to evaluate the extraction efficiency of this method. Briefly, 1 g of shellfish sample was weighed into a 15 mL centrifuge tube and extracted by vortex mixing in 3 mL 100% methanol for 3 min. The mixture was then centrifuged at 2500× *g* for 10 min. The extract solutions were mixed well and aliquots were stored in amber glass vials at −20 °C until analyzed by LC-MS/MS and other screening methods.

### 4.4. Tissue Extract Hydrolysis

The tissue extracts, from both EU and Jellett extraction methods, were hydrolyzed using a modified method published by Mountfort *et al.* [23]. A 125 µL aliquot of a 2.5 N NaOH solution was mixed with 1 mL of the extract and heated at 76 °C for 40 min in a tightly sealed 1.5 mL vial (Sun Brokers, Inc., Wilmington, NC, USA) to avoid sample loss due to evaporation. The hydrolyzed sample was neutralized with 125 µL of a 2.5 N HCl solution and filtered through a 0.22 µm PTFE syringe filter (Fisher Scientific, Pittsburgh, PA, USA). Samples were analyzed by LC-MS/MS immediately after hydrolysis or stored at 4 °C and analyzed by rapid methods within 2 days of hydrolysis. Total toxin levels (free plus esterified) were calculated with correction for the 25% volume increase from the additions of base and acid.

### 4.5. LC-MS/MS

Shellfish extracts, from both the EU and Jellett methods, were analyzed by Ultra-performance Liquid Chromatography (UPLC; Acquity system, Waters Co., Milford, MA, USA) coupled with a triple quadrupole tandem mass spectrometer (MS/MS, ABSciex 5500, Framingham, MA, USA). For each sample, 10 µL of filtered extract was injected into the UPLC-MS/MS. The UPLC was equipped with a 0.2 µm pre-filter followed by a 2.1 × 4 mm C8 Security guard cartridge and a 2.1 × 100 mm, 5 µm Luna C8 reverse phase column (Phenomenex, Torrance, CA, USA). The acidic chromatographic conditions used in this study were described in the EU-Harmonised method [35] and by McCarron *et al.* [36]. A description of the mobile phases, linear gradients, run time and analyte detection are described in detail in Trainer *et al.* [15].

The analytes were quantified with individual seven-point external calibration curves prepared in methanol from certified reference standards (CRM-OA-c, CRM-DTX-1-c, CRM-DTX-2-c and CRM-YTX-b) purchased from National Research Council Canada (Halifax, Nova Scotia). A linear best fit resulted in a correlation coefficient of *R* = 0.99 for each curve. Standard calibration curve ranges were as follows: OA: 0.71 to 286 ng/mL; DTX-2: 0.44 to 177 ng/mL; DTX-1: 0.72 to 289 ng/mL; YTX: 0.56 to 224 ng/mL A water blank and a check vial containing all 4 analytes was run after every 10 to 12 samples as quality control. The % relative standard deviation for OA = 2.2%, DTX-2 = 1.3%, DTX-1 = 2.5% and YTX = 3.3%. Each extract was run in the hydrolyzed and non-hydrolyzed form. Hydrolysis reduces esterified compounds to the parent form and allows the detection of total toxin for OA, DTX-2, and DTX-1. Final reporting was in µg/100 g tissue for shellfish extracts. The limits of detection (LOD) and limits of quantitation (LOQ) in shellfish tissue for monitored toxins were, respectively: OA, 10 pg on column and 1.25 µg/100 g tissue; DTX-1, 10 pg on column and 1.25 µg/100 g tissue; and DTX-2, 8 pg on column and 1.00 µg/100 g tissue.

### 4.6. Enzyme-Linked Immunosorbent Assay (ELISA)

The DSP ELISA test is an immunoassay based on the recognition of OA and its isomers by rabbit anti-OA antibodies, produced by Abraxis (DSP ELISA kit, PN 52001; Warminster, PA, USA). Detailed instructions on its use can be found at the manufacturer’s website [37]. This test can detect OA in shellfish and water samples; however, while the antibodies react with 100% affinity to OA, they have only 50% cross-reactivity to DTX-1, DTX-2. The antibody reaction is initiated by adding shellfish sample or standard and OA-HRP conjugate to a solution of the anti-OA antibodies. The OA antibodies are then incubated with a secondary antibody (goat anti-rabbit) immobilized on the surface of the 96-well microplate. The concentration of DSP toxins in a sample or standard is inversely proportional to the intensity of the color signal produced by 3,3′,5,5′-tetramethylbenzidine (TMB) substrate and determined by interpolation from OA standard curve constructed with each analysis. The test has a 5-point standard curve ranging from 0.1 to 5.0 ppb OA in solution. The limit of detection for OA by this test is 10 μg/100 g shellfish tissues.

### 4.7. Protein Phosphatase 2A (PP2A)

Protein-phosphatase inhibition assays were developed about a decade ago based on the finding that serine/threonine protein phosphatases are inhibited by OA group of toxins. Of the four major classes of this group of enzymes, PP1, PP2A, PP2B and PP2C, PP2A has the highest affinity for OA [38]. Commercially available PP2A test kits (OkaTest, ZEU, Spain) were developed using purified human protein phosphatase type 2A and p-nitrophenylphosphate (pNPP) as a colorimetric substrate in a 96-well microplate format to measure OA and its analogs including DTX-1, DTX-2 and DTX-3. Dinophysistoxin-3, acyl ester form of OA, DTX-1 or DTX-2, is converted to its free form by alkaline hydrolysis of the methanolic extracts. Hydrolyzed sample extracts were diluted with assay buffer and incubated with enzyme phosphatase solution at 30 °C. Toxins in sample extracts are directly proportional to the color product, p-nitrophenol (p-NP) formed in the reaction between the uninhibited enzyme and pNPP substrate that can be measured at 405 nm using a microplate reader. Total DST concentration was determined in hydrolyzed shellfish extract based on a 5-point calibration standard curve, ranging from 0.5 to 2.0 nM. LOD and LOQ for blank mussel are 4.4 and 5.6 μg/100 g, respectively [29].

### 4.8. Jellett Rapid Test Strip

A rapid field test kit was developed to screen for DSTs in shellfish samples by Jellett Rapid Testing, Ltd. (Nova Scotia, Canada). This test is based on the anti-OA antibodies used in the ELISA method and was developed for simple field application. The Jellett Rapid Test for DSP is a lateral flow immuno-chromatographic (LFI) system similar to the pregnancy test strips. In the strip format, the three major DSP toxins (OA, DTX-1, DTX-2) are detected with similar affinity with the 50% reduction in color intensity of the test line set at 5 nM to trigger a positive result compared to the control line [20]. Hydrolyzed extracts were diluted with a running buffer solution supplied with the test kit and 100 µL of the diluted sample was applied to the sample pad of a test strip. After 30 min, the test and control lines were fully developed and the color intensity was compared to a color guide supplied by the manufacturer. If the T(est) line was lighter than the C(ontrol) line, the result was positive; if it had the same color intensity as the C line, then the result was negative (Figure 4).

## 5. Conclusions

In this study, the applicability of screening assays for the determination of DSTs in shellfish tissues was compared to the standard regulatory LC-MS/MS method. Few other studies have explored the effectiveness of screening assays using naturally contaminated samples, particularly those that contain high concentrations of DTX-1 and acyl derivatives, collectively called DTX-3. Good agreement was observed between the PP2A test and LC-MS/MS, however, when toxin levels exceeded the upper working range of the assay, the PP2A test underestimated total DTX-1 compared to LC-MS/MS values. The few false negatives (8%) observed with the PP2A test might be eliminated by choosing a conservative threshold value (e.g., 12 μg/100 g) for screening. The ELISA showed relatively high false positives (>20%) and no false negatives. The Jellett rapid test gave >30% false negative results due to the incomplete extraction efficiency of the method used as recommended by the manufacturer. Finally, our results suggest that PP2A is the screening test of choice for Washington State monitoring program as it may provide a safety buffer for the harvest of large amounts of shellfish prior to confirmatory regulatory testing, or may suffice as a tool to maintain an open status of the shellfish beds. However, further screening tests must be conducted over the next few years using a variety of subsistence, recreational, and commercial shellfish from the region in order to determine the most effective scenarios for their use. In Washington State, we envision that the implementation of a strategy where monitoring for the presence of *Dinophysis* species together with the application of toxin screening would allow shellfish harvesters to avoid unnecessary disposal of harvested product and eliminate costly recalls.
